# Stretta radiofrequency treatment vs Toupet fundoplication for gastroesophageal reflux disease: a comparative study

**DOI:** 10.1186/s12876-020-01310-2

**Published:** 2020-05-27

**Authors:** Lifeng Ma, Tao Li, Guochao Liu, Jianlong Wang, Zhaoqiang Yin, Jiansheng Kang

**Affiliations:** Department of General Surgery, the Second Hospital of Hebei Medical University, 19 Jiuzhong Street, Shijiazhuang, 050000 China

**Keywords:** Gastroesophageal reflux, Stretta radiofrequency procedure, Toupet fundoplication, Survival analysis, Prognosis

## Abstract

**Background:**

Outcomes of gastroesophageal reflux disease (GERD) using Toupet fundoplication (TF) and Stretta radiofrequency (SRF) have not been compared and this study was conducted to compare therapeutic efficacy of the two methods.

**Methods:**

This retrospective study analyzed a total of 230 patients undergoing TF or SRF at our hospital. Baseline data, reflux symptoms, the DeMeester scores, lower esophageal sphincter (LES) pressure and adverse events were compared over 1 year period.

**Results:**

A total of 226 patients were included in the study. The time and frequency of reflux and percentage of reflux time before and 12 months after therapy were not significantly different. There were significantly interactions between the therapy method and follow-up time on the DeMeester score and LES pressure. Twelve months post therapy, the DeMeester score was significantly higher in SRF than in TF group, while the LES pressure was lower. At 12 months after therapy, multivariate Cox proportional regression analysis showed that reflux frequency, the DeMeester score and LES pressure were risk factors for poor prognosis in TF group, while reflux frequency and the DeMeester score, and LES pressure were risk factors for poor prognosis in SFR group.

**Conclusions:**

Compared with TF, SFR can significantly improve the esophageal pH and pressure in GERD patients without increasing the risk of poor prognosis.

## Background

Gastroesophageal reflux disease (GERD) is a gastrointestinal motility disorder that results from the reflux of stomach contents into the esophagus or oral cavity resulting in symptoms or complications [1, 2]. The disease is predominant in men aged 40 to 60, with typical symptoms of heartburn and reflux [3, 4]. With the improvement of living standards and the change of people’s eating habits, the incidence of GERD is still high and rising year by year, particularly in North America and East Asia [5–7]. Due to slow onset and progress of the disease, it is often ignored, resulting in a large number of patients having esophageal stricture, non-cardiogenic chest pain, asthma, aspiration pneumonia and other adverse complications. As a consequence, medication is often ineffective and surgery is required to treat severe or stubborn GERD [8, 9]. Currently, laparoscopic surgical fundoplication remains the gold standard treatment for GERD that is refractory to medical management, but it may cause abdominal distension, recurrence, gastric ulcer and other complications [10, 11]. Although heartburn and regurgitation are less after surgery, a large proportion of patients still need to use anti-reflux medication after surgical fundoplication [7].

In the past decade, with the progress of minimally invasive treatments, SRF is being increasingly used to treat refractory GERD [12, 13]. The Stretta device uses a balloon-tipped four-needle catheter that delivers radiofrequency energy into the smooth muscle of the esophagogastric junction (EGJ). In the first report in 2001, the Stretta procedure was shown to be a promising procedure in 25 GERD patients [14]. Over the last 17 years this therapeutic modality has been markedly improved and has been used in more than 20,000 patients [7]. SRF is shown to be safer to the muscular layer and mucosa, but less effective in anti-reflux than the traditional method [15] and does not provide long-term symptom control [16]. Despite numerous studies, there is no consensus as which method is better for GERD treatment, although a recent systematic review, that compared the Stretta procedure to sham in four RCTs, has found that the procedure is not more efficacious than sham intervention [17], although the procedure is effective in reducing use of proton pump inhibitor [18].

In this study, we compared the therapeutic effect and prognosis of the two surgical methods for patients diagnosed with GERD. The findings would help better planning of clinical treatment for GERD patients.

## Methods

### Patients

A total of consecutive 230 patients diagnosed with GERD and underging TF (*n* = 142) and SRF (*n* = 88) at our hospital between January 2014 and June, 2017 were enrolled in the retrospective study. GERD was diagnosed by endoscopy showing esophagitis or abnormal esophageal pH, a DeMeester score ≥ 14.7 with symptom correlation of ≥50%, and/or > 73 reflux episodes during 24-h ambulatory impedance monitoring period, lower than normal LES pressure by esophageal manometry. They were endoscopically confirmed to have Los Angeles grade A or B esophagitis, with non-hiatal hernia or small (< 2 cm) hiatal hernia. Patients were included if they were 18 years old or older, had clinical symptoms such as regurgitation, retrosternal pain and heartburn. Patients were excluded if they had diseases in the central nervous system or connective tissue, were previously performed esophageal or gastric surgery. Patients with esophageal stricture, shortened esophagus, impaired distal esophageal peristalsis, autoimmune diseases, collagen vascular diseases, Barrett’s esophagus, coagulation disorders, acute heart failure, cardiogenic shock and other important organ diseases and previous thoracic surgery were also excluded. Patients who had medications that affect the secretion of gastric acid and gastrointestinal motility within 7 days were also excluded to avoid the impact of the medication on evaluation of the therapeutic effect.

Patient demographics and clinical data at the time of diagnosis, including age, gender, duration of typical GERD symptoms prior to diagnosis, past and present medications, dietary inhibits, smoking, and clinical symptoms of typical GERD, were collected.

The study protocols were approved by the Ethical Review Committee of Hebei Medical University (Approval no HMU-2212, Nov, 2013) and conform to the ethical guidelines of the 1975 Declaration of Helsinki. Written consent was obtained from each patient included in the study.

### Treatment

TF was performed under general anesthesia. The type of TF was either all laparoscopic or open or mix. After laparotomy, the abdominal esophagus was isolated. The left and right cruses of diaphragm were sutured for 1–2 sutures to close the esophageal hiatus. One cm gap was left between the top first suture and the esophagus to allow the patency of esophagus. Then, the gastric fundus was folded at 270 degrees and fixed with 2–3 sutures at the gastric fundus and on both sides of esophagus.

For SRF, the procedure was performed according to Triadafilopoulos [19]. Briefly, during a deeply sedated esophagogastroduodenoscopy (EGD), the eligibility was confirmed and the distance of the squamo-columnar junction (used as the approximate location for the gastroesophageal junction) was measured. A guide wire was introduced to the duodenum through the EGD, and the EGD was then withdrawn. A RF delivery catheter (Kanglian Medical Equipment Co., Ltd., Beijing) from a radiofrequency device (S500L, CURON MEDICAL Inc., USA) was inserted orally using the guide wire. The Stretta catheter is made up with an inflatable and flexible balloon-basket with four electrode needles. The balloon was inflated when it was 2 cm proximal to the squamo-columnar junction to deploy the electrode needles (22-gauge, 5.5-mm length). RF energy was delivered for 60 s. The needles were then withdrawn. After the balloon was deflated, and the catheter was rotated 45° to delivery radiofrequency. This process is repeated every 0.5 cm to cover the area 2 cm above and 1.5 cm below the squamo-columnar junction and six sets below the cardias for a total of 14 sets of needle deployments.

### Outcome assessment

Reflux time and frequency, the DeMeester score, LES pressure, esophageal pH and prognosis were recorded, measured and analyzed. GERD relapse was the primary endpoint. The DeMeester score was measured as described previously [20]. A DeMeester score of > 14.7 was regarded as having reflux. LES pressure was measured using XDJ-S8S Esophageal Motility System (Kelly Photoelectrics Technology Co., Ltd., Hefei, China) according to the manufacturer’s instructions. Medications such as anti-cholinesterase drugs and acid-suppressing agents that may affect esophageal motor function and secretion of gastric acid were discontinued 7 days before the assessment. Patients were fasting for 4 to 6 h before the pressure measurement. Esophageal pH was measured using Digitrapper pH-Z Recorder with combined pH-Impedance (*Given* Imaging Ltd., USA) by inserting calibrated pH electrodes from the nasal cavity to 5 cm above LES. The esophageal pH at three meals, standing position and lying position was recorded for 24 h to calculate the DeMeester score. If the pH was < 4 and DeMeester score was greater than 14.72, it was regarded as acid reflux. The outcome was classified as good or poor prognosis. The poor prognosis included events such as dysphagia, abdominal distention, diarrhea, chronic stomach pain and recurrence of GERD. The patients were followed up for 1 year at 2 month intervals.

### Statistical analysis

Data were analyzed with SPSS 17.0 software (SPSS Inc., Chicago, IL, United States) and were presented as the mean ± SD for continuous variables and as percentages and proportions for categorical variables. For the statistical analyses, the Kolmogorov-Smirnov test was used to assess the normality data. Independent *t*-test was applied to compare the means between two groups. Two-factor repeated measures ANOVA was used to compares the means between repeated measurement data. Counting data were expressed as percentage and tested using *X*^2^ test or the Fisher’s exact probability method. The stepwise Cox regression procedures were used to analyze the risk factors affecting prognosis. A value of *P* value < 0.05 was considered statistically significant.

## Results

### Baseline characteristics

At the end of follow-up, two patients each were lost in the TF group and the SRF group. As a result, 140 patient in the TF group and 86 patients in the SRF group were analyzed. Mean age was 53.7 (± 6.1) and 62.8% were males. Mean duration of GERD was 12.3 (± 7.3) years and the mean body mass index (BMI) was 28.2 ± 8.6. There were no difference in the gender, age and BMI between the two groups. 71% patients had chronic comorbid conditions. The most common comorbidity was hypertension (30.9%) followed by coronary heart disease (23.8%) and diabetes mellitus (16.3%). However, the percentages of patients with these comorbidities were not different between the groups (Table 1).

**Table 1:** Comparison of baseline data between gastroesophageal reflux disease patients undergoing Toupet fundoplication and the Stretta procedure

Surgery	No. patients	Gender (male/female)	Age (year)	body mass index	GERD family history [*n* (%)]	Hypertension [*n* (%)]	Coronary disease [*n* (%)]	Diabetes [*n* (%)]
Toupet fundoplication	140	90/50	54.8 ± 5.9	29.2 ± 11.9	21 (15.0)	40 (28.6)	36 (25.7)	21 (15.0)
Stretta procedure	86	52/34	52.6 ± 6.2	27.2 ± 10.9	16 (19.6)	30 (34.9)	18 (20.9)	16 (18.6)
*χ*^2^ (*t*)		0.318	0.793^a^	0.993^a^	0.226	0.916	0.645	0.023
*P* value		0.619	0.479	0.879	0.526	0.339	0.422	0.858

^a^ denotes *t* value

### Reflux symptoms

Before and 12 month after GERD treatments, the mean time and frequency of reflux, and percentage of reflux time were not significantly different between the two groups (*P* > 0.05, Table 2).

**Table 2:** Comparison of reflux status between gastroesophageal reflux disease patients after Toupet fundoplication and the Stretta procedure

Surgery	No. patients	Reflux time (h)		Reflux frequency		Percent of reflux time (%)	
		Before	12 months	Before	12 months	Before	12 months
Toupet fundoplication	140	2.6 ± 1.8	1.7 ± 1.4	127.4 ± 54.5	30.7 ± 15.2	9.8 ± 4.0	5.8 ± 2.1
Stretta procedure	86	2.8 ± 1.9	2.0 ± 1.5	131.7 ± 59.5	33.2 ± 16.8	*10*.1 ± 4.6	6.1 ± 2.5
*t*		1.566	0.918	0.582	0.841	0.392	1.771
*P* value		0.107	0.390	0.670	0.496	0.871	0.359

### DeMeester score and LES pressure

Before, 2 and 12 months after GERD treatments, the DeMeester score and LES pressure had significantly interactions over the treatment and time (*P* < 0.05), and there were significant main effects on the DeMeester score and LES pressure by the treatment and follow-up time (*P* < 0.05). The DeMeester score was significantly higher in the SRF group than in the TF group (8.8 vs 7.3. *P* < 0.05), while LES pressure was the opposite (11.6 vs 12.8, *P* < 0.05, Table 3). However, at 2 months after operation, these parameters were similar between the groups (*P* > 0.05, Table 3).

**Table 3:** Comparison of DeMeester score and lower esophageal sphincter pressure between gastroesophageal reflux disease patients after Toupet fundoplication and the Stretta procedure

Surgery	No. patients	DeMeester score			LES pressure (mmHg)		
		Before	Two months	12 months	Before	Two months	12 months
Toupet fundoplication	140	27.6 ± 14.1	11.5 ± 6.1	7.3 ± 4.4	7.9 ± 3.2	11.5 ± 3.0	12.8 ± 3.1
Stretta procedure	86	28.9 ± 13.2	11.9 ± 7.3	8.8 ± 5.0^a^	8.0 ± 3.6	10.8 ± 3.4	11.6 ± 3.3^a^
*F* value		*F*_int_ = 14.524, *F*_surgery_ = 3.892, *F*_time_ = 15.480			*F*_int_ = 12.514, *F*_surgery_ = 3.982, *F*_time_ = 14.380		
*P* value		*P*_int_ < 0.001, *P*_surgery_ = 0.011, *P*_time_ < 0.001			*P*_int_ = 0.015, *P*_sugery_ = 0.001, *P*_time_ < 0.001		

^a^*P* < 0.05 vs Toupet fundoplication

### Adverse events

The overall percentages of poor prognosis as measured by adverse events were 21.4% (30/140) in the TF group and 11.6% (10/86) in the SRF group, which were not significantly different (*P* > 0.05, Table 4). The incidences of dysphagia, abdominal distension, diarrhea, chronic stomach pain and recurrence of GERD were not different significantly between the two groups (*P* > 0.05, Table 4), although there were two relapse GERD patients in SRF group.

**Table 4:** Comparison of poor prognosis between gastroesophageal reflux disease patients after Toupet fundoplication and the Stretta procedure (n, %)

Toupet fundoplication	No. patients	Dysphagia	Bloating	Diarrhea	Chronic stomach pain	GERD relapse	
Stretta procedure	140	8 (5.7)	8 (5.7)	6 (4.3)	6 (4.3)	2 (1.4)	
Toupet fundoplication	86	2 (2.3)	4 (4.7)	2 (2.3)	2 (2.3)	0	
*χ*^2^ (*t*)		0.620	0.043	0.187	0.167	–	
*P* value		0.486	0.866	0.792	0.792	0.744	

### Risk factors

Factors resulting in poor prognosis and adverse events were analyzed using multivariate Cox proportional regression using prognosis after treatment as dependent variable (good prognosis = 0, and poor prognosis =1). Time and frequency of reflux, and percentage of reflux time, the DeMeester score and LES pressure at the 1 year mark were included in the analysis. The results showed that for TF patients, high reflux number [RR = 1.701, 95% CI (1.929, 3.981), *P* = 0.035], DeMeester score [RR = 1.867, 95% CI (1.232, 2.370), *P* = 0.001] and low LES pressure [RR = 1.399, 95% CI (1.909, 2.196), *P* = 0.0347] were risk factors for poor prognosis. Similarly, high reflux frequency [RR = 1.581, 95% CI (1.168, 2.145), *P* = 0.022] and the DeMeester score [RR = 1.899, 95%CI (1.522, 2.658), *P* = 0.004)], and low LES pressure (RR = 1.856, 95% CI (1.565, 4.677), *P* = 0.015) were risk factors for poor prognosis in SFR group (Tables 5 and 6).

**Table 5:** Multivariate Cox proportional regression analysis of prognostic factors in the Toupet fundoplication

Variable	*B*	*SE*	Wald *χ*^2^ value	*P* value	*RR*	95% *CI*
Reflux time	0.051	0.104	1.212	0.872	1.062	(0.574, 1.967)
Reflux fequency	0.174	0.215	5.220	0.035	1.701	(1.929, 3.981)
Percentage of acid reflux	0.178	0.218	2.183	0.625	1.102	(0.672, 1.587)
DeMeester score	0.549	0.136	6.466	0.001	1.867	(1.232, 2.370)
LES pressure	0.364	0.213	5.138	0.047	1.399	(1.909, 2.196)

**Table 6:** Multivariate Cox proportional regression analysis of prognostic factors in the Stretta procedure

Variable	*B*	*SE*	Wald *χ*^2^ value	*P* value	*RR*	95% *CI*
Reflux time	0.018	0.123	0.395	0.694	1.010	(0.720, 1.419)
Reflux fequency	0.499	0.135	6.123	0.022	1.581	(1.168, 2.145)
Percentage of reflux time	−0.419	0.315	2.213	0.883	0.661	(0.351, 1.255)
DeMeester score	0.593	0.128	7.489	0.004	1.898	(1.522, 2.658)
LES pressure	0.513	0.870	7.160	0.017	1.856	(1.565, 4.677)

## Discussion

GERD is a common digestive disease with typical symptoms of heartburn and regurgitation. It is regarded as an important public health issue. In the past decade, a number of clinical treatments have been developed for GERD. Among them, proton pump inhibitors (PPIs) are regarded as the most effective medication for GERD, due to their profound and consistent acid suppression ability and have become the main treatment of GERD. However, after discontinuation of drug, the recurrence rate is high and long-term medication compliance has been a burden for patients [21]. Poor compliance, lack of adherence to correct time of PPI administration and incorrect diagnosis are some of the important hurdles that plague successful treatment of GERD patients in clinical practice [22]. Surgery and radiofrequency therapy could be considered for patients who are not interested, concerned about, developed adverse events and who are unable to comply with regular, 1 year medical treatment.

Several studies have shown that laparoscopic TF is more effective than Nissen fundoplication to relieve the symptom with less risk of complication [23]. SRF has been shown to be effective in improve the symptoms and quality of life of GERD patients, and may be more effective and safer than TF for GERD [12]. However, the side by side comparison of therapeutic effect and long-term outcome of the two methods are rare and a recent study shows that both methods are safe and effective for the control of GERD-related extra-esophageal symptoms and the reduction of PPI use [15]. If laparoscopic and open surgery are compared, laparoscopic surgery was shown to have better short-term outcome, but long-term outcomes were similar for GERD patients [24].

Our analysis showed that the time and frequency of reflux and acidic reflux time are similar between the two techniques before and 1 year after the treatment, suggesting that SRF has similar therapeutic effect as compared to traditional TF. Hu et al. showed that SRF improves the reflux barrier of LES, as a result of reduced transient LES relaxation due to the ablation or demodulation of vagus afferent fiber nerve near the sphincter [25, 26]. TF is shown to reduce acid exposure and increase LES pressure to improve GERD symptoms [27]. As a consequence of similar mechanism, it is expected that the two methods would have similar short-term therapeutic effect as observed in our study.

DeMeester scores and LES pressures before, 2 and 12 months after the treatment showed that they change over the follow-up time and treatment methods, displaying significant time-related main effects on the two parameters. The DeMeester score at 12 month after surgery was higher in the SRF group as compared with these in the TF group, while the LES pressure was the opposite, suggesting that SRF is less effective to increase the esophageal pH and more effective to reduce the LES pressure in GERD patients. This result is not consistent with early results that the patients in the LTF group were more satisfied with their quality of life than those in the Stretta procedure group (*P* < 0.05) [15]. The mechanism underlying these differences between the two procedures might be due to different cellular and tissue responses and is worthy investigation.

The overall incidence of poor prognosis (adverse events) were statistically similar between the TF and the SRF groups. In addition, the incidences of dysphagia, abdominal distension, diarrhea, chronic stomach pain and recurrence of GERD were not different significantly between the two groups, indicating that the long-term prognosis of the two methods are similar for GERD treatment.

To analyze the risk factors of poor prognosis and their impact, multivariate Cox proportional regression was performed for the two groups. The results showed that the reflux frequency, DeMeester score and LES pressure are significantly correlated to poor prognosis in both groups. It may be related to the mode of action and the purpose of treatment. Both techniques achieve the therapeutic effect through reducing frequency and severity of GERD-related extraesophageal symptoms and decreasing PPI use. Since the reflux frequency, DeMeester score and LES pressure are related to poor prognosis, it is highly recommended that the patients are regularly examined for these parameters to better evaluation of long-term efficacy and outcome after surgery.

However, there are limitations in this study. It was single-center study with limited number of participants and relatively short follow-up time. The many adverse reactions and events may have not been included and only a few factors have been analyzed for their impact on prognosis. Further large scale and multiple-center studies and longer follow-up are needed to validate our conclusions.

## Conclusions

Taken together, we have found that SFR can significantly improve the esophageal pH and pressure in GERD patients without increasing the risk of poor prognosis and are equally effective and safe as compared to TF. Therefore, SFR could be an option for the treatment of refractory GERD.

## Data Availability

The datasets used during the current study are available from the corresponding author on reasonable request.

## Abbreviations

GERD: Gastroesophageal reflux disease
SRF: Stretta radiofrequency
LES: Lower esophageal sphincter
EGJ: Esophagogastric junction
BMI: Body mass index
TF: Toupet fundoplication
